# Cigarette smoke-associated inflammation impairs bone remodeling through NFκB activation

**DOI:** 10.1186/s12967-021-02836-z

**Published:** 2021-04-21

**Authors:** Yi Lu, Yuanpu Peter Di, Ming Chang, Xin Huang, Qiuyan Chen, Ni Hong, Beth A. Kahkonen, Marissa E. Di, Chunyan Yu, Evan T. Keller, Jian Zhang

**Affiliations:** 1 School of Medicine, Southern University of Science and Technology, No. 1088 Xueyuan Blvd, Nanshan District, Shenzhen, 518055 Guangdong China; 2 Guangdong Provincial Key Laboratory of Cell Microenvironment and Disease Research, Shenzhen, 518055 Guangdong China; 3 Department of Environmental and Occupational Health, University of Pittsburgh, 100 Technology Dr, Pittsburgh, PA 15261 USA; 4 State Key Laboratory of Oncology in South China, Collaborative Innovation Center for Cancer Medicine, Sun Yat-Sen University Cancer Center, Guangzhou, Guangdong China; 5 Department of Urology & Pathology, University of Michigan, Ann Arbor, MI 48109 USA; 6 Department of Urology, University of Pittsburgh, Pittsburgh, 15260 USA

**Keywords:** Cigarette smoking, Inflammation, Bone remodeling, NFκB

## Abstract

**Background:**

Cigarette smoking constitutes a major lifestyle risk factor for osteoporosis and hip fracture. It is reported to impair the outcome of many clinical procedures, such as wound infection treatment and fracture healing. Importantly, although several studies have already demonstrated the negative correlation between cigarette consume and impaired bone homeostasis, there is still a poor understanding of how does smoking affect bone health, due to the lack of an adequately designed animal model. Our goal was to determine that cigarette smoke exposure impairs the dynamic bone remodeling process through induction of bone resorption and inhibition of bone formation.

**Methods:**

We developed cigarette smoke exposure protocols exposing mice to environmental smoking for 10 days or 3 months to determine acute and chronic smoke exposure effects. We used these models, to demonstrate the effect of smoking exposure on the cellular and molecular changes of bone remodeling and correlate these early alterations with subsequent bone structure changes measured by microCT and pQCT. We examined the bone phenotype alterations in vivo and ex vivo in the acute and chronic smoke exposure mice by measuring bone mineral density and bone histomorphometry. Further, we measured osteoclast and osteoblast differentiation gene expression levels in each group. The function changes of osteoclast or osteoblast were evaluated.

**Results:**

Smoke exposure caused a significant imbalance between bone resorption and bone formation. A 10-day exposure to cigarette smoke sufficiently and effectively induced osteoclast activity, leading to the inhibition of osteoblast differentiation, although it did not immediately alter bone structure as demonstrated in mice exposed to smoke for 3 months. Cigarette smoke exposure also induced DNA-binding activity of nuclear factor kappaB (NFκB) in osteoclasts, which subsequently gave rise to changes in bone remodeling-related gene expression.

**Conclusions:**

Our findings suggest that smoke exposure induces RANKL activation-mediated by NFκB, which could be a “smoke sensor” for bone remodeling.

**Supplementary Information:**

The online version contains supplementary material available at 10.1186/s12967-021-02836-z.

## Introduction

Cigarette smoking (CS) has been identified as a key contributing factor in the development of a variety of fatal and debilitating disorders, including respiratory and cardiovascular diseases. It is also confirmed that CS contributes to delayed bone union during fracture healing [1]. Epidemiological evidence indicates that CS enhances bone loss and fragility fracture incidence, making it a major lifestyle risk factor for osteoporosis. The association between CS and fracture has been reported in both men and women [2–5]. However, the underlying mechanisms of how CS negatively affects bone are not completely known, partially obfuscated by the influence of smoking on sex hormones in both genders [6]. Specifically, decreased bone mineral density (BMD) has been shown to correlate with increased fracture risk [7–10]. The persistent evidence of higher levels of bone resorption markers in smokers [11] and reduced serum osteocalcin levels in early postmenopausal women smokers [12, 13] suggests that an imbalance in the bone remodeling process arises in smoking individuals.

Normal bone development and maintenance are sustained through a balanced communication between osteoclasts and osteoblasts, cells responsible for bone resorption and bone formation, respectively, in accordance with mechanical demands that keep the shape and strength of bone within strict limits [14]. This dynamic balance is coordinated through cell–cell contact, and also tightly controlled by a variety of signals including hormones, cytokines, and mechanical stimuli [15]. In this way, intercellular communications initiate and coordinate the bone remodeling cycle. Inversely, the imbalance of bone formation and bone resorption leads to pathological bone loss, which subsequently results in bone-related diseases such as osteoporosis [16].

Smoking is not only harmful to an individual’s own health but also surrounding subjects. Secondhand smoke (SHS) is an important risk factor for non-smoker women suffering from lung cancer [17]. Furthermore, CS also increased the risk of hip fracture in smokers compared to never smokers [4]. However, supporting evidence in animal models to directly link cigarette smoke exposure with dynamic changes in bone remodeling is still lacking. Specifically, the cellular and molecular mechanisms responsible for the adverse effects of both CS and SHS on bone remain poorly understood due to lack of suitable animal experimental models. Despite the suggestion that smoking has a direct adverse effect on skeletal remodeling and bone cells, the observation of impaired bone formation was during nicotine exposure in an animal model, rather than showing a direct causal association with using cigarette smoke, which contains complex chemical mixtures of more than nicotine alone. Recently, there is a report that showed in an in vivo mouse model that animals exposed for 6 months to cigarette smoke has dramatically affect bone integrity and biomechanical properties by using the ApoE knockout mouse model [18].

The aim of the present study was to explore the effect of CS on the dynamic balance between bone resorption and bone formation using mouse models systematically exposed to a smoking environment. We compared smoking-associated defects of animals with an exposure time of 10 days and 3 months using two protocols to compare the effects of acute and chronic smoke exposure on animals, respectively. Osteoblast formation and bone resorption were investigated among smoke and non-smoke (filtered air) exposed animals. Biochemical analyses using cigarette smoke extract (CSE) in vitro further identified the DNA-binding activity of transcription factors regulating changes in bone remodeling-related gene expression.

## Materials and methods

### Reagents and antibodies

Research-grade 3R4F cigarettes were obtained from the University of Kentucky Tobacco Research and Development Center (Lexington, KY, USA). Recombinant human M-CSF (rhM-CSF) and rhRANKL were purchased from R & D Systems (Minneapolis, MN, USA). NFκB P65 antibodies and Cyclin D1 antibodies were purchased from Santa Cruz Biotechnology (Santa Cruz, CA, USA). QNZ (EVP4593) was purchased from Selleckchem (Houston, TX, USA). All chemical reagents were purchased from Sigma (St. Louis, MO, USA).

### Mice

Eight-week-old Fvb/n mice (The Jackson Laboratory, Bar Harbor, ME, USA) were housed under pathogen-free conditions in accordance with NIH guidelines. All animal experiments were conducted in accordance with approved by the Institutional Animal Care and Use Committee at the University of Pittsburgh. The mice were treated with whole body cigarette smoke exposure in a smoking chamber with an average monitored smoke exposure concentration of 100.3 ± 7.2 mg/m^3^ total smoke particulate (TSP). In protocol 1, the chronic smoke exposure model, the mice (n = 20) were assigned into two groups: non-smoke and smoke. The mice in the smoke group were exposed to cigarette smoke for 6 h/day and 5 days/week for 12 weeks. In protocol 2, the acute smoke exposure model, the mice were divided into 4 groups: non-smoke, smoke, non-smoke with lipopolysaccharide (LPS), and smoke with LPS (n = 12–14/group). The smoke mice were exposed for 6 h/day and 5 days/week for 2 consecutive weeks, and the LPS mice were treated with 0.2 mg/kg body weight/mouse via intranasal instillation. The mice were allowed to grow for the indicated time until sacrifice. Evidence of bone changes caused by smoke exposure was evaluated using a micro computed tomography (microCT), peripheral quantitative computed tomography (pQCT), histology, and bone histomorphometry.

### microCT analysis

The fifth lumbar vertebrae of the mice were fixed in 3.7% formaldehyde and used for 3D microCT analysis. First, 2D CT horizontal slices of the lumbar were obtained with a Scanco vivaCT40 (Scanco Medical, Bassersdorf, Switzerland). The thickness of the slices was 21 μm. Three-dimensional images were reconstructed using software provided by Scanco. The trabecular bone was measured in the tissue area. Parameters analyzed from 3D images included the ratio of trabecular bone volume and total bone volume (BV/TV), trabecular thickness (Tb.Th), trabecular number (Tb.N), and trabecular separation (Tb.Sp).

### Bone mineral density analysis

Tibiae from all animals were measured for bone mineral density (BMD) using pQCT (Stratec, Dundas, Ontario, CA). pQCT scans from three slices of the proximal metaphysis region were performed to obtain total bone mineral densities and trabecular bone mineral densities.

### Histopathology and bone histomorphometry

Histopathology was performed as described previously [19]. Briefly, bone specimens (tibiae) were fixed in 10% formalin for 24 h, then decalcified using 10% EDTA for 6 days. The specimens were then paraffin-embedded, sectioned (4 µm), and stained with hematoxylin and eosin (H&E) to assess histology. Histomorphometric analysis was performed on H&E stained sections and a variety of parameters were quantified using Bioquant Osteo II (Bioquant Image Analysis Corp, Nashville, TN) as previously reported. The terminology used is as recommended by the Histomorphometry Nomenclature Committee of the American Society for Bone and Mineral Research. The sections were used for tartrate-resistant acid phosphatase (TRAP) staining (acid phosphatase kit, model 387-A; Sigma Diagnostics, Livonia, MI, USA). For TRAP staining, the specimens were stained with acid phosphatase and tartrate solution for 1 h at 37 °C. The area of TRAP positivity was quantified using Bioquant Osteo II software. Four discontinuous random regions of interest were examined within each tibia. TRAP-positive cells were counted along the endocortical bone surfaces. The number of TRAP-positive cells is reported as osteoclasts/mm. Osteoblasts were identified as cuboidal-shaped cells on the bone surface. The number of osteoblasts/mm bone was calculated by averaging the numbers of osteoblasts in five randomly chosen fields.

### Preparation of CSE

Research-grade 3R4F cigarettes were smoked using a peristaltic pump (VWR International, West Chester, PA, USA) [20]. Briefly, each cigarette was smoked for 10 min, until a 17 mm butt remained. Four cigarettes were bubbled through 40 ml of cell growth medium, and this solution, regarded as 100% strength CSE, was adjusted to a pH of 7.4. Following preparation of CSE, the solution was filtered through 0.2-µm filters immediately prior to treat cells. The highest concentrations of CSE used in our experiments were chosen such that cellular viability was no less than 95% of that in control cells cultured in the absence of CSE.

### Cell culture

MC3T3-E1 subclonal 4 (MC-4) cells were kindly provided by Dr. Guozhi Xiao (University of Pittsburgh, Pittsburgh, PA, USA). MC-4 cells were pre-osteoblast, expressing osteoblast phenotypic marker genes and mineralizing only after growth in AA (ascorbic acid)-containing medium [21]. MC-4 cells were maintained in AA-free α-MEM (Invitrogen, Carlsbad, CA, USA), supplemented with 10% fetal bovine serum and 1% penicillin/streptomycin.

### Mouse bone morrow mononuclear cell (MBMC) culture

Primary mouse bone marrow cells were obtained by flushing femora from 8-week old Fvb/n mice or from the mice at the end of treatments. The bone marrow cells were incubated in α-MEM medium (Invitrogen, Carlsbad, CA) with 20% FBS (HyClone, Pittsburgh, PA, USA) and 1% penicillin/streptomycin (Invitrogen) overnight at 37 °C in 100-mm tissue culture plates to allow for separation of non-adherent and adherent cells.

### Osteoclast formation and bone resorption assay

Osteoclast formation assays were performed by culturing 1 × 10^5^/well non-adherent bone marrow cells in 96-well plates in 0.1 ml of α-MEM with 10% FBS for 7 days. Cells were incubated with recombinant mouse M-CSF (10 ng/ml), RANKL (50 ng/ml), and CSE at concentrations indicated. Half of the media was changed at days 4 and 7. After 10 days of culture, the cells were fixed with 2% formaldehyde and stained with the K-ASSAY TRAP staining kit (Kamiya Biomedical, Seattle, WA). Positively stained cells that contained 3 or more nuclei were counted as osteoclast-like multinucleated cells. Analysis of all osteoclast formation experiments included data from 3 independent experiments.

To perform the bone resorption pit assay, murine non-adherent bone marrow cells (1 × 10^5^/well) were seeded on dentin slices in 96-well plates and treated as above. After 2 weeks of culture, osteoclasts on dentin slices were confirmed by TRAP staining, and bone resorption lacunae were stained with hematoxylin. The mean area of resorption was determined microscopically with SPOT software (Diagnostic Instruments, Inc. Sterling Heights, MI).

### Osteoblast differentiation

MC-4 cells or primary murine bone marrow stromal cells (mBMSC) were plated in 6-well plates at 5 × 10^4^ cells/cm^2^ and incubated in differentiation medium, which contains growth medium and ascorbic acid (AA, 50 μg/ml). They were treated as indicated for 5–7 days. Then cells were provided with inorganic phosphate for the mineralization assay, or cell lysates were collected for detection of alkaline phosphatase (ALP) activity, and the supernatants were applied for measurement of TNFα levels, or total RNA was extracted for determination of osteoblast differentiation marker gene expression levels. To confirm CSE induced NF-κB pathway activation, mBMSCs were treated with QNZ, NF-κB inhibitor, at 1–20 nM,w/o 1% CSE and the supernatant was collected and TNFα levels were measured by ELISA.

### Mineralization assay

Mineralization analysis was performed by von Kossa staining and/or Alizarin red (AR-S) staining. Briefly, for von Kossa staining, the cells were rinsed once with PBS, fixed with 95% ethanol for 15 min at 37 °C, and then serially hydrated in 80%, 50%, 20% ethanol to distilled water (ddH_2_O). The water was removed, a 5% silver nitrate solution was added to each well, and the plate was covered with Al foil and incubated for 1 h at 37 °C. After the silver nitrate solution was removed, the plate was rinsed twice with ddH_2_O, exposed to bright light for at least 30 min, then rinsed with water, dehydrated in 50%, 95%, 100% ethanol, and dried for image analysis. For alizarin red (AR-S) staining, the cells were rinsed once with PBS, fixed with cold 70% ethanol for 60 min, and rinsed 3 times with ddH_2_O to remove ethanol completely. The cells were then stained with 40 mM AR-S (pH 4.2) at RT for 15 min with rotation on a shaker. After that, cells were rinsed with ddH_2_O five times to remove unbound AR-S and one time with PBS for 15 min to further reduce non-specific staining. Photographs were then taken at this point. To quantify the AR-S stain, 10% cetylpyridinium chloride (CPC) was added to the wells at RT for 30 min with shaking. Aliquots of these AR-S extracts were taken, diluted in 10% CPC solution, and the concentrations were determined by absorbance measurement at 562 nm on a 96-well multiple plate reader.

### Quantitative real-time RT-PCR

Total RNA was extracted from the differentiated osteoclast, osteoblast, or MC-4 cells at the end of treatments using TRIzol reagent (Life Technologies, Gaithersburg, MD) according to the manufacturer’s protocol. Reverse transcription (RT) was performed using 2 µg of total RNA and 100 pmol of random hexamers (Applied Biosystems, Foster, CA) in a total volume of 20 µl containing 12.5 U MultiScribe reverse transcriptase (Applied Biosystems) according to the manufacturer’s instruction. PCR was performed on iCycler iQ multicolor real-time PCR detection system (Bio-Rad, Hercules, CA) using SYBR Green PCR kit (Applied Biosystem). Primer sequences used in this study are listed in Table 1. All the amplifications were performed as follows: initial denaturation at 95ºC for 10 min followed by 45 cycles of 95ºC for 15 s and 60ºC for 60 s. Melting curve analysis was performed to evaluate the purity of the PCR products. Triplicate samples were run for each primer set. The target gene expression was calculated relative to GAPDH (a housekeeping control gene) using the ΔCT method as previously described [22].

**Table 1:** Primer sequences used for quantitative RT-PCR

Gene	Forward primer (5′ to 3′)	Reverse primer (5′ to 3′)
Cat K	GTGTTGGTGGTGGGCTATG	GCAGGCGTTGTTCTTATTCC
TRAP	CACCCTGAGATTTGTGGCTGT	CGGTTCTGGCGATCTCTTTG
CTR	GGCGACTATCTACTGCTTCTG	GATTCCGTGGTTCCTGATGG
Runx2	TGGCTTGGGTTTCAGGTTAGGG	TCGGTTTCTTAGGGTCTTGGAGTG
OCN	TAGTGAACAGACTCCGGCGCTA	TGTAGGCGGTCTTCAAGCCAT
Col I	CTGACTGGAAGAGCGGAGAG	GCACAGACGGCTGAGTAGG
OSX	AGAGGTTCACTCGCTCTGACGA	TTGCTCAAGTGGTCGCTTCTG
BSP	AAGAGGAAGAAAATGAGAACGA	GCTTCTTCTCCGTTGTCTCC
OPN	CCA ATG AAA GCC ATG ACC ACA	CGT CAG ATT CAT CCG AGT CCA C
OPG	TACCTGGAGATCGAATTCTGCTT	CCATCTGGACATTTTTTGCAAA
RANKL	GAAACTCACAGCCCTCTCTCTTG	GCATCGGAATACCTCTCCCAATC
GAPDH	CAGTGCCAGCCTCGTCCCGTAGA	CTGCAAATGGCAGCCCTGGTGAC

### Nuclear protein extraction

Nuclear protein extractions from the cells were prepared by using Nuclear Extract Kit (Active Motif, Carlsbad, CA) according to the manufacturer's protocol. Briefly, the cells were washed twice with ice-cold PBS and phosphatase inhibitors. Cells were removed with a cell lifter by gentle scraping, and cell pellets were collected after centrifugation for 5 min at 500*g*. The pellets were resuspended in 1 × hypotonic buffer and centrifuged at 14,000*g* for 30 s at 4 °C. From the resuspension, the supernatant was collected as the cytoplasmic fraction. The insoluble pellet, which contained nuclei, was resuspended in 50 µl Complete Lysis Buffer and vortexed for 15 s, incubated and suspended for 30 min on ice on a rocking platform set at 150 rpm, then vortexed again for 30 s and centrifuged at 14,000*g* for 20 min at 4 °C. The supernatant (nuclear fraction) was aliquoted into pre-chilled tubes stored at -80 °C. Protein concentration was determined with the BCA protein assay kit (Pierce, Rockford, IL).

### Electrophoretic mobility shift assays (EMSA)

Detection of protein-oligonucleotide complexes was performed with an EMSA gel-shift kit (Active Motif). Briefly, NF-κB oligonucleotide probes (Santa Cruz Biotechnology) were labeled with [-^32^P] ATP at 50,000 cpm/ng using T4 polynucleotide kinase. Nuclear extracts (5 µg) were incubated with 1 µg of poly(deoxyinosinic–deoxycytidylic acid), gel shift reaction buffer [10 mM Tris (pH 7.5), 50 mM NaCl, 1 mM DTT, 1 mM EDTA, and 5% glycerol], and 0.5 ng of labeled oligonucleotide probes for 30 min at room temperature. For supershifts with p65 antibody or Cyclin D1 antibody, nuclear extracts were pre-incubated with 1 µl of p65 antibody or cyclin D1 antibody (Santa Cruz Biotechnology) for 15 min at room temperature before the addition of binding buffer and probe. DNA–protein complexes were resolved by electrophoresis through a 4% polyacrylamide gel containing 50 mM Tris (pH 7.5), 0.38 M glycine, and 2 mM EDTA. The gel was then dried and visualized by autoradiography.

### ELISA

Quantikine Mouse TNFα/TNFSF1A Immunoassay kits were purchased from R & D Systems (Minneapolis, MN). The levels of TNFα in bone marrow plasma collected from both animal models, and in the supernatants collected from CSE-treated BMSC cells, were measured using the ELISA kit according to the manufacturer’s instructions. The sensitivity of this assay is 0.51 pg/ml.

### Alkaline phosphatase (ALP) activity

The cell lysates were harvested in 1 × Passive Lysis Buffer (Promega, Madison, WI) and clarified by centrifugation at 13,000*g* for 20 min at 4ºC. Five microliters of cell lysate was added to each well (96-well plate) containing 150 μl p-nitrophenyl phosphate (pNPP) (Sigma) at 37 °C for 10–60 min depending on the level of ALP activity in the extracts. ALP activity was determined by absorbance measurement at 405 nm on a 96-well plate reader. ALP activity was normalized to total protein.

### Statistics

Statistical significance was determined for multivariate comparisons using ANOVA and Fisher’s probable least significant difference for post hoc analysis. Student’s *t* test was used for bivariate analyses. Statistical significance was determined as P < 0.05. Statistical calculations were performed using Statview software (Abacus Concepts, Berkeley, CA).

## Results

### Study design

In this study, we hypothesize that cigarette smoke exposure impairs the dynamic bone remodeling process through induction of bone resorption and inhibition of bone formation. The overall aims of this study were to (i) investigate smoke exposure impairs the bone remodeling through induction of bone resorption and inhibition of bone formation in vivo, (ii) determine the mechanisms of how smoke exposure affects bone remodeling in vitro at both cellular and molecular levels. To address these aims, we used in vivo animal models, to demonstrate the effect of smoking exposure on the cellular and molecular changes of bone remodeling and correlate these early alterations with subsequent bone structure changes measured by microCT and pQCT. We established smoking mice model, including 3-month smoking mice to mimic chronic smoke exposure, and 10-day smoking mice to mimic the acute smoke exposure. We examined the bone phenotype alterations in vivo and ex vivo in the acute and chronic smoke exposure mice by measuring bone mineral density and bone histomorphometry. Next, we measured osteoclast and osteoblast differentiation gene expression levels in each group. The function changes of osteoclast or osteoblast were evaluated. Finally, we tested cigarette smoke induces DNA-binding activity of NFκB using cigarette smoke extract (CSE). In all experiments, the sample size was determined by previous experience of the statistical variance encountered, statistical calculations were made based on the following criteria: power of 80% and a significance level < 0.05.

### Chronic smoke exposure enhances osteoclast maturation and bone resorption-related gene expression

**Fig. 1:**
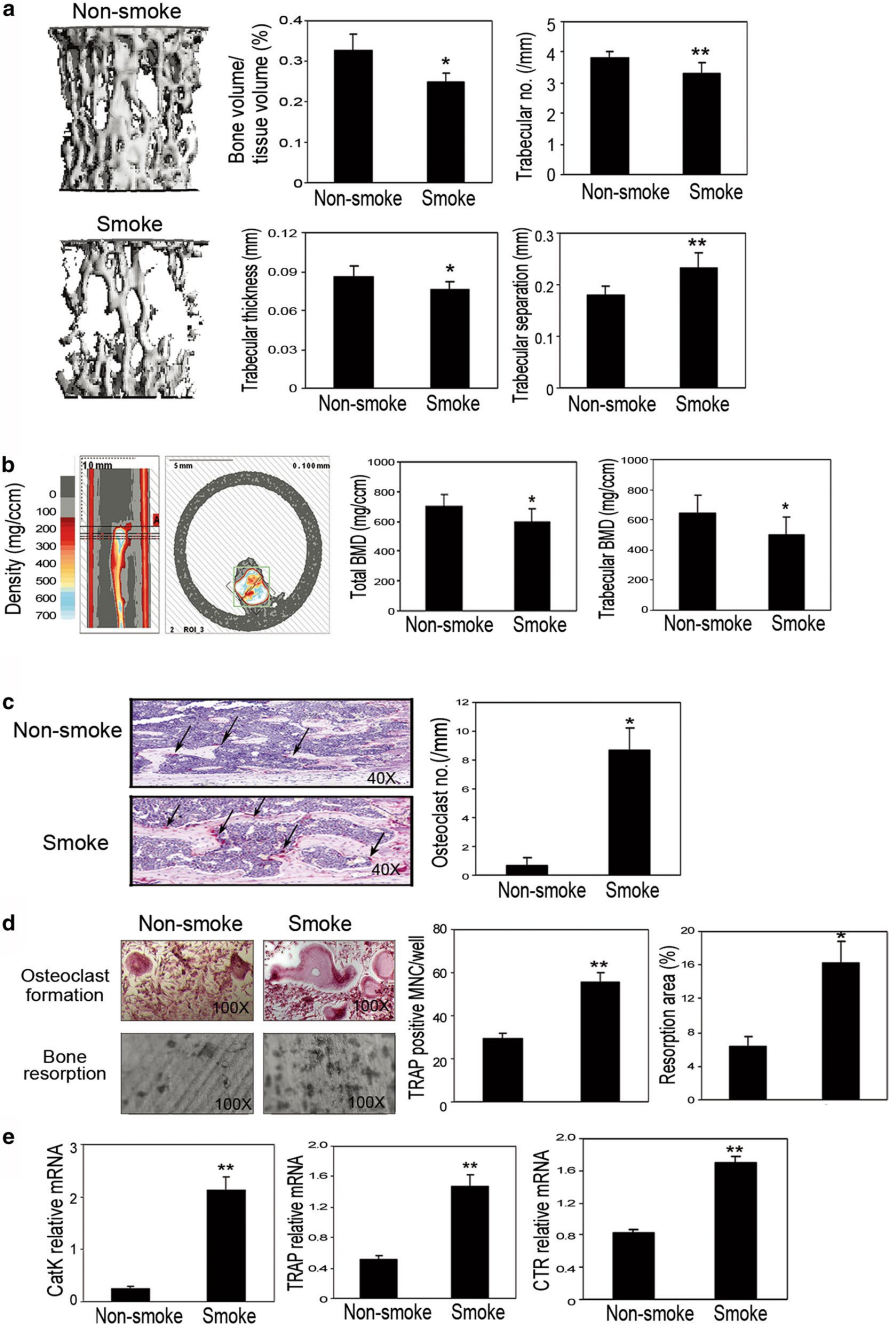
Chronic cigarette smoke exposure caused bone loss in mouse model both in vivo and ex vivo. **a** microCT of the epiphyseal region of the fifth lumbar spine (L5). (Left) Three-dimensional images of microCT. (Right) The parameters of bone phenotype were determined by microCT, including bone volume vs tissue volume (BV/TV), trabecular numbers (Tb. N), trabecular thickness (Tb. Th), and trabecular separation (Tb. Sp). **b** BMD and trabecular BMD were measured by pQCT in the tibiae. (Upper panel) Slices were scanned by pQCT. The small box in the right panel indicates the tibia. The reference slice and the slices examined are indicated on a representative tibia. (Lower panel) Graph of the average bone density obtained by pQCT in tibiae from non-smoke and smoke groups. **c** Histological and histomorphometric analyses of the tibiae. (Left) TRAP staining on decalcified tibiae slides. (Right) Number of osteoclasts on bone surface as measured by bone histomorphometry analysis. **d** Ex vivo osteoclast formation and bone resorption assay were performed using the non-adherent bone morrow cells. Number of osteoclast-like multinucleated cells per well was quantified. Samples were evaluated in quadruplicate. Bone resorption area was determined microscopically with SPOT software. Samples were evaluated in triplicate. Results are reported as mean ± SD. **e** Gene expression of bone resorption markers including Cat K, TRAP, and CTR were examined by real-time RT-PCR. Specific mRNA expression was calculated relative to the expression of GAPDH using the ΔCT method. Bars are indicated mean ± SE. Data is representative of three independent experiments. ^*^P < 0.05 compared to non-smoke group; ^**^P < 0.001 compared to non-smoke group

For all the experiments, 8-week-old Fvb/n mice were exposed to whole body cigarette smoke in a smoking chamber. To study the effect of smoke exposure on bone development, animals were passively exposed to cigarette smoke for 6 h/day and 5 days/week for 12 weeks in protocol 1. Based on previous studies suggesting that the spine is more severely affected by smoking than other sites [23, 24], as spine contains predominantly trabecular bone, bone structural changes were initially captured by microCT in lumbar spine L5. Smoke exposure significantly decreased bone volume, trabecular number, trabecular thickness, and increased trabecular separation, as determined by microCT analysis (Fig. 1a). The smoke exposure also decreased total BMD and trabecular BMD in the tibiae (Fig. 1b) as determined by pQCT. On the tibiae slides stained with TRAP, the effective induction of TRAP-positive osteoclast formation was analyzed in the smoke-exposed animals compared to their non-smoke-exposed counterparts (Fig. 1c), indicating that smoke exposure induced osteoclast activity in bone in vivo. Consistent with this result, the number of osteoclasts was significantly higher in the smoke-exposed animals than in the non-smoke-exposed animals as determined by bone histomorphometric analysis (Fig. 1c).

We also performed an ex vivo osteoclast formation assay using non-adherent bone marrow cells isolated from these animals, aiming to determine the cellular and molecular changes caused by smoke exposure. Smoke exposure significantly induced osteoclast formation and thus bone resorption in vitro, an observation that is further supported by increased numbers of TRAP-positive cells and resorption area (Fig. 1d). To ascertain the molecular alterations of bone resorption, we studied the gene expression of bone resorption markers, such as cathepsin K (CatK), TRAP, as well as calcitonin receptor (CTR). The expression levels were elevated in the cultured non-adherent bone marrow cells from the smoke-exposed animals compared to expression levels in the non-smoke-exposed animals, as determined by real-time RT-PCR (Fig. 1e). These results suggest that smoke exposure does indeed induce gene expression of bone resorption markers in animals.

### Acute smoke exposure and LPS administration induce osteoclast formation

**Fig. 2:**
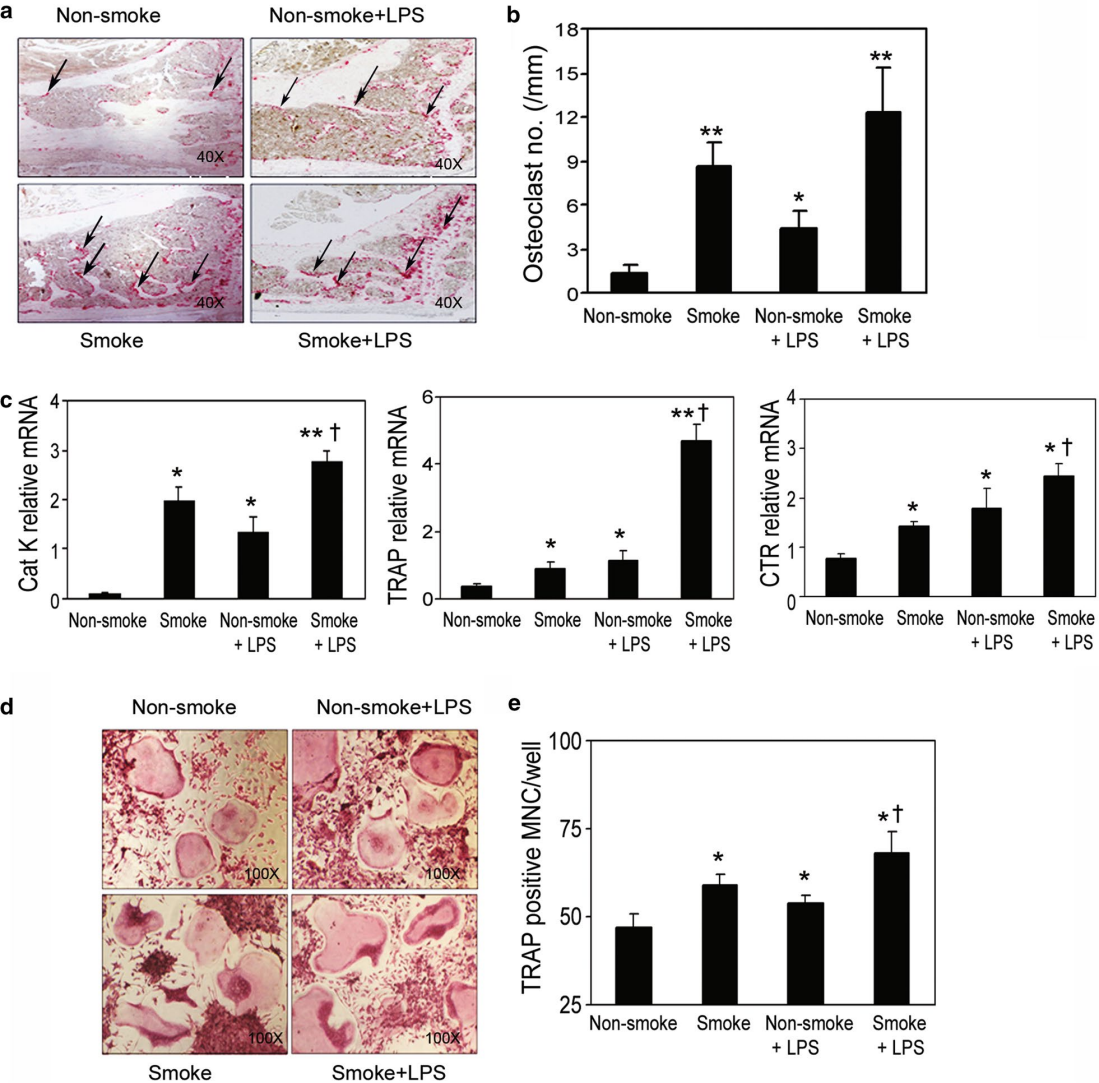
Enhanced induction of osteoclast activity through acute cigarette smoke exposure with LPS administration. **a** TRAP staining on the decalcified tibiae slides. **b** Number of osteoclasts on the bone interface was measured by bone histomorphometry. **c** Non-adherent bone marrow cells from the indicated test groups were seeded in 6-well plates, then cultured for 7 days. Gene expression of the osteoclast formation markers was detected by real-time RT-PCR. **d** Osteoclast formation was performed using non-adherent bone marrow cells and stained by TRAP in vitro. **e** Quantification of TRAP-positive multinucleated cells per well. Samples were evaluated in quadruplicate. Results are reported as mean ± SD. ^*^P < 0.05 compared to non-smoke group; ^**^P < 0.001 compared to non-smoke group; ^†^P < 0.05 compared to non-smoke with LPS group

The short-term cigarette smoke exposure paradigm was tested according to the acute and passive smoking situations in protocol 2, in which the smoke exposure was carried out for 6 h/day, 5 days per week for 2 consecutive weeks. Animals were divided into 4 groups: non-smoke, smoke, non-smoke with LPS at 5 μg/mouse intranasal instillation, and smoke with LPS (n = 12–14/group). The LPS groups were included for 2 purposes: first, to serve as a positive control to demonstrate osteoclast activation [25] and concurrent inhibition of osteoblast differentiation [26]; second, to test whether smoke exposure would enhance the adverse response of bone in the presence of inflammation. In order to test how smoke exposure affects the bone structure, bone changes on L5 were examined by microCT, and total and trabecular BMD in tibiae was examined by pQCT. Compared to the animals that were exposed to cigarette smoke for 3 months, the animals with the short-term smoke exposure of 10 days did not, in fact, have immediately altered bone structure (Additional file 1: Fig. S1). However, on TRAP-stained tibiae slides, the induction of TRAP-positive osteoclast formation was observed in the smoke-exposed animals and not in the non-smoke-exposed animals (Fig. 2a, b), indicating that cellular activation of osteoclasts may occur prior to structural changes in vivo. The mRNA expression levels of bone resorption markers CatK, TRAP, and CTR were greatly elevated in the non-adherent bone marrow cells that were isolated and cultured from the smoke-exposed animals (Fig. 2c). In addition, smoke exposure combined with LPS administration further enhanced the gene expression of those same markers compared to either the smoke-exposed animals without LPS administration or the non-smoke-exposed animals with LPS administration (Fig. 2c).

We also explored the effect of short-term smoke exposure on osteoclastogenesis. Non-adherent bone marrow cells were isolated from the aforementioned experimental mice and tested for osteoclast formation. Smoke exposure significantly induced in vitro osteoclast formation in the cells isolated from smoke-exposed animals, compared to those from the non-smoke-exposed animals (Fig. 2d, e). LPS treatment was observed to amplify the effects of smoke exposure. This suggests that under inflammatory conditions, smoke exposure may contribute to enhanced osteoclast formation. In support of the findings described above, LPS and nicotine were reported to stimulate osteoclast formation in osteoclast precursor RAW 264.7 cells [25] and primary non-adherent bone marrow cells through direct enhancement of receptor activator of NF-kappaB ligand (RANKL) expression [27] and suppression of Osteoprotegerin (OPG) expression [28, 29]. A decoy receptor homolog for RANKL, OPG inhibits receptor activator of NF-kappaB (RANK) by binding to RANKL. OPG is thus tightly involved in regulating NF-κB activation [30]. Continuous exposure to cigarette smoke also increased the number of inflammatory cells, including alveolar macrophages, as well as led to the recruitment of neutrophils, which are commonly associated with LPS-induced inflammation [31]. Taken together, our results suggest that adverse effects on bone may occur rapidly and the underlying cellular and molecular changes may precede the measurable bone structural alterations.

### Smoking exposure inhibits osteoblast differentiation and activityW

**Fig. 3:**
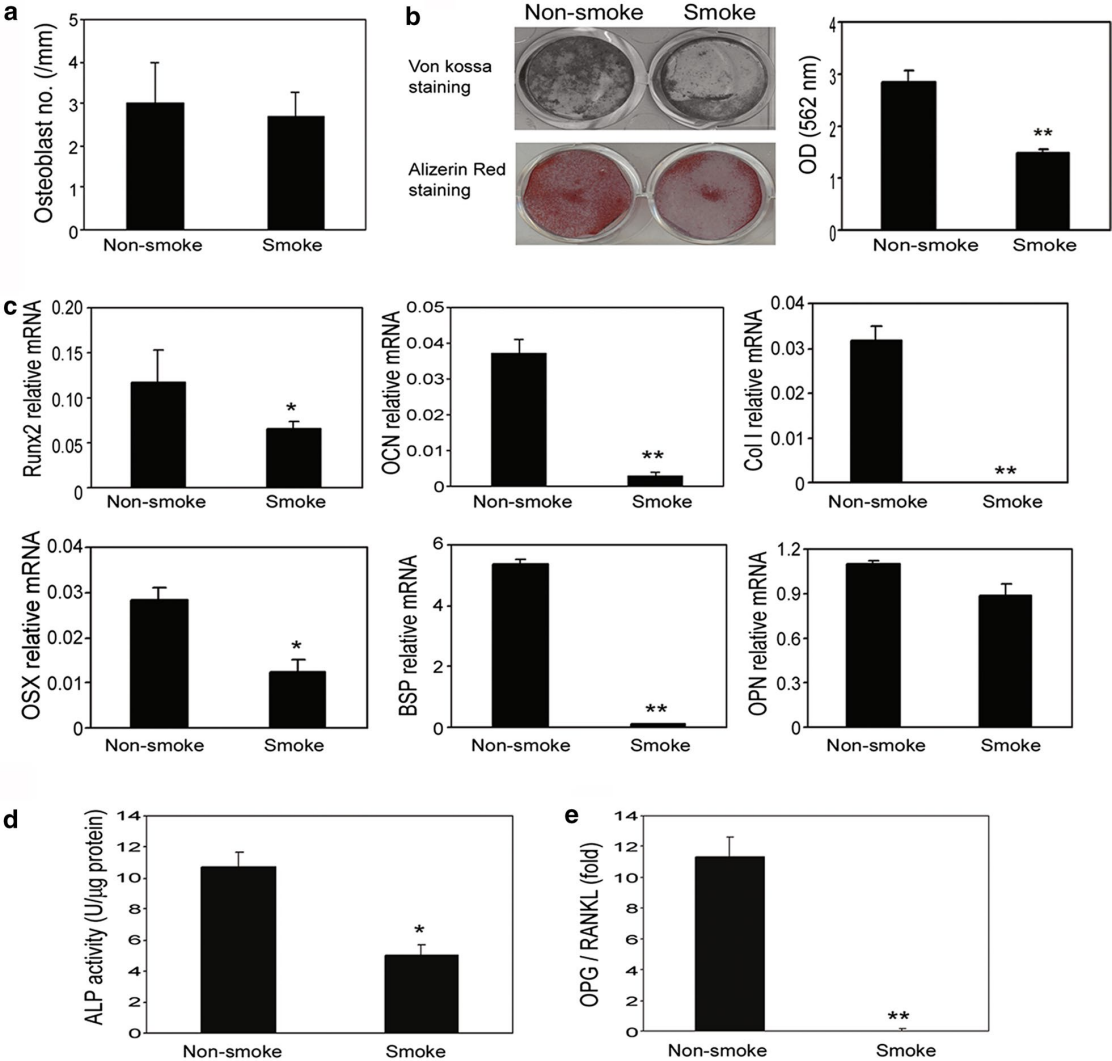
Chronic cigarette smoke exposure inhibited bone mineralization and downregulated the gene expression of osteoblast differentiation markers. **a** Smoke exposure did not alter the number of osteoblasts on bone interface measured by histomorphometry in the protocol 1 mice model. **b** BMSCs were seeded at 5 × 10^4^ cells/cm^2^ in 6-well plates and given differentiation medium for 10 days. Inorganic phosphate (5 mM) was added to the culture medium for another 4 days for mineralization assays. The capacity of bone mineralization was measured by von Kossa staining and alizarin red staining. **c** Gene expression of osteoblast differentiation markers including Runx2, OCN, Col I, OSX, BSP and OPN was determined by real-time RT-PCR. Levels of Runx2, OCN, Col I, OSX, BSP, and OPN mRNA were normalized to GAPDH. **d** ALP activity was normalized to total protein. **e** OPG and RANKL gene expression in the osteoblasts were examined by quantitative RT-PCR. The ratio of OPG to RANKL is illustrated. Data are representative of three independent experiments. Data are presented as the mean ± SD from triplicates. ^*^P < 0.05 compared to non-smoke group; ^**^P < 0.001 compared to non-smoke group

**Fig. 4:**
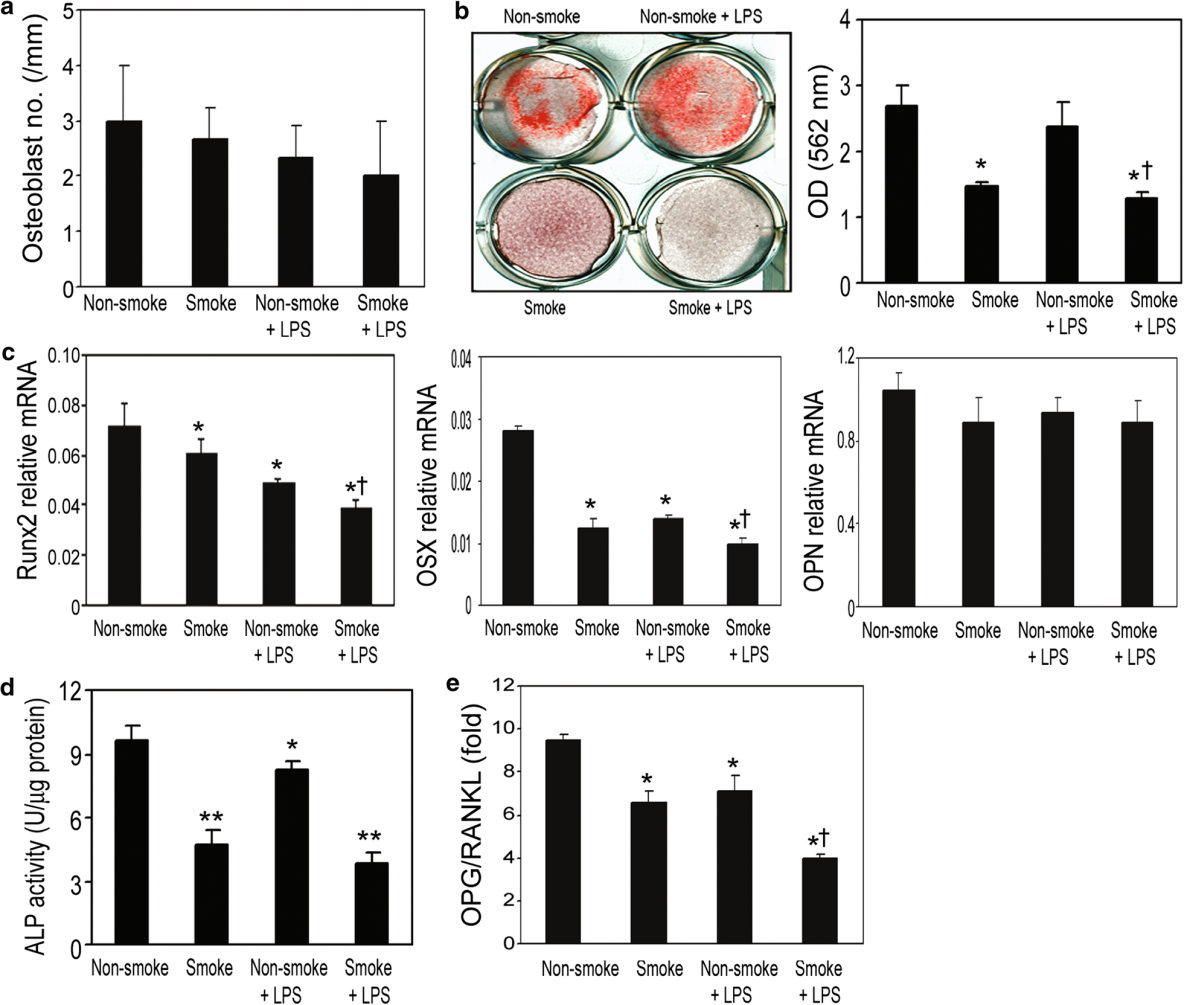
Acute cigarette smoke exposure diminished osteoblast-specific gene expression and bone mineralization at cellular and molecular levels, with additive effects when alongside LPS administration. **a** Smoke exposure did not alter the number of osteoblasts on the bone interface as measured by histomorphometry in the protocol 2 mice model. **b** The capacity of bone mineralization was measured by alizarin red staining. **c** Gene expression of osteoblast differentiation markers including Runx2, OSX and OPN was determined by real-time RT-PCR. Levels of Runx2, OSX, and OPN mRNA were normalized to GAPDH. **d** ALP activity was normalized to the total protein level. **e** OPG and RANKL gene expression in the differentiated BMSCs were examined by quantitative RT-PCR. The ratio of OPG to RANKL was calculated. Data are presented as the mean ± SD from triplicates. ^*^P < 0.05 compared to non-smoke group; ^**^P < 0.001 compared to non-smoke group; ^†^P < 0.05 compared to non-smoke with LPS group

To investigate the effects of smoke exposure on osteoblast differentiation, the number of osteoblasts on the bone surface in tibiae was quantified by histomorphometry. We found that in both animal protocols, smoke exposure failed to alter the number of osteoblasts in the animals (Figs. 3a and 4a). However, at the cellular and molecular levels, in vitro osteoblast differentiation and bone mineralization were inhibited in the smoke-exposed animals compared to in the non-smoke-exposed animals, as measured by both in vitro silver nitrate (von Kossa) and Alizarin Red-S (AR-S) staining (Figs. 3b and 4b).

Next, we performed real-time RT-PCR to determine the gene expression of osteoblast differentiation markers runt-related transcription factor 2 (RUNX2), osteocalcin (OCN), type I collagen (Col I), osterix (OSX), bone sialoprotein (BSP), and osteopontin (OPN) using RNA isolated from these cells. Smoke exposure significantly reduced expression levels of RUNX2, OCN, Col I, OSX and BSP, except OPN (Figs. 3c and 4c). ALP activity in the cell lysates from the smoke-exposed mice was significantly inhibited compared to that of the non-smoke-exposed animals (Figs. 3d and 4d). Smoke exposure reduced the ratio of OPG/RANKL in these cells (Figs. 3e and 4e). Due to technical shortcomings, we were not able to measure the in vivo bone formation rate in this study, but nevertheless, our results clearly suggest that smoke exposure inhibited osteoblast differentiation and bone mineralization in vitro. Similar to results from protocol 2 settings, additive effects of smoke exposure with LPS administration were also observed using the indicated parameters (Fig. 4). These results indicate that under inflammatory conditions, smoke exposure may further enhance the inhibitory effect on osteoblast differentiation and bone mineralization.

### Cigarette smoke extract (CSE) impairs bone remodeling mediated by the NFκB pathway

**Fig. 5:**
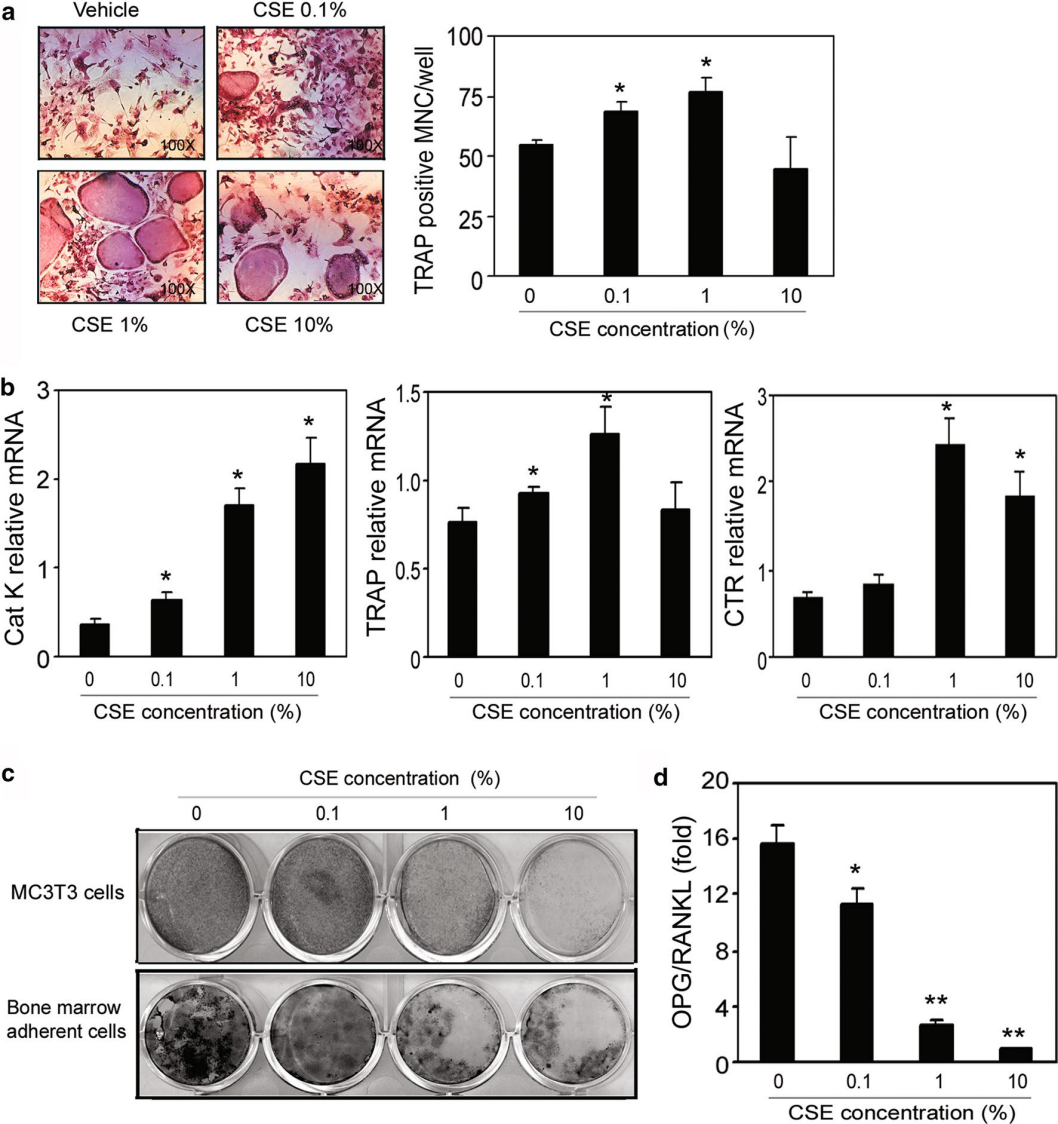
The reciprocal effects of CSE on bone remodeling mediated by RANKL-dependent pathway. **a** Non-adherent bone morrow cells collected from Fvb/n mice were placed into 96-well plates and treated with M-CSF and RANKL in the presence or absence of CSE at indicated concentrations for 7 days, then stained for TRAP. (Left) TRAP-positive multinucleated cells indicate osteoclast formation. (Right) Quantification of TRAP-positive multinucleated cells per well. CSE dose-dependently increased the number of osteoclast-like multinucleated cells. **b** CSE treatments induced osteoclast-specific gene expression in a dose-dependent manner. Samples were evaluated in quadruplicate. Results are reported as mean ± SD. **c** BMSCs and MC-4 cells were placed in 6- or 12-well plates and cultured in differentiation medium with the indicated concentrations of CSE for 10 days, and inorganic phosphate (5 mM) was added to the culture medium for the last 4 days. The capacity of bone mineralization was measured by von Kossa staining. CSE dose-dependently inhibited bone mineralization in both MC3T3 cells and BMSCs. **d** Total RNA was extracted from differentiated BMSCs, and RANKL and OPG gene expression were determined by real-time RT-PCR. The ratio of OPG to RANKL expression was dramatically reduced by CSE. All the data are from three independent experiments

Studies of adverse effects of cigarette smoke exposure on bone have been made challenging by the properties of cigarette smoke, which is composed of over 4,000 different chemicals, including 43 known carcinogenic compounds and 400 other toxins. For example, polycyclic aromatic hydrocarbons present in cigarette smoke were shown to cause loss of bone mass and bone strength in rats [32]. Therefore, CSE was chosen in the present study to investigate the mechanisms of smoke exposure’s adverse effects on bone. Surprisingly, CSE induced TRAP-positive, multinucleated osteoclast-like cell formation in non-adherent bone marrow cells (Fig. 5a). Consistent with this result, CSE also induced the mRNA expressions of CatK, TRAP, and CTR (Fig. 5b). In MC3T3 cells (a commonly used murine osteoblast cell line) and murine bone marrow adherent cells, CSE significantly inhibited bone mineralization in vitro (Fig. 5c). At the molecular level, the OPG/RANKL ratio of mRNA expression was decreased dose-dependently in both cells (Fig. 5d).

**Fig. 6:**
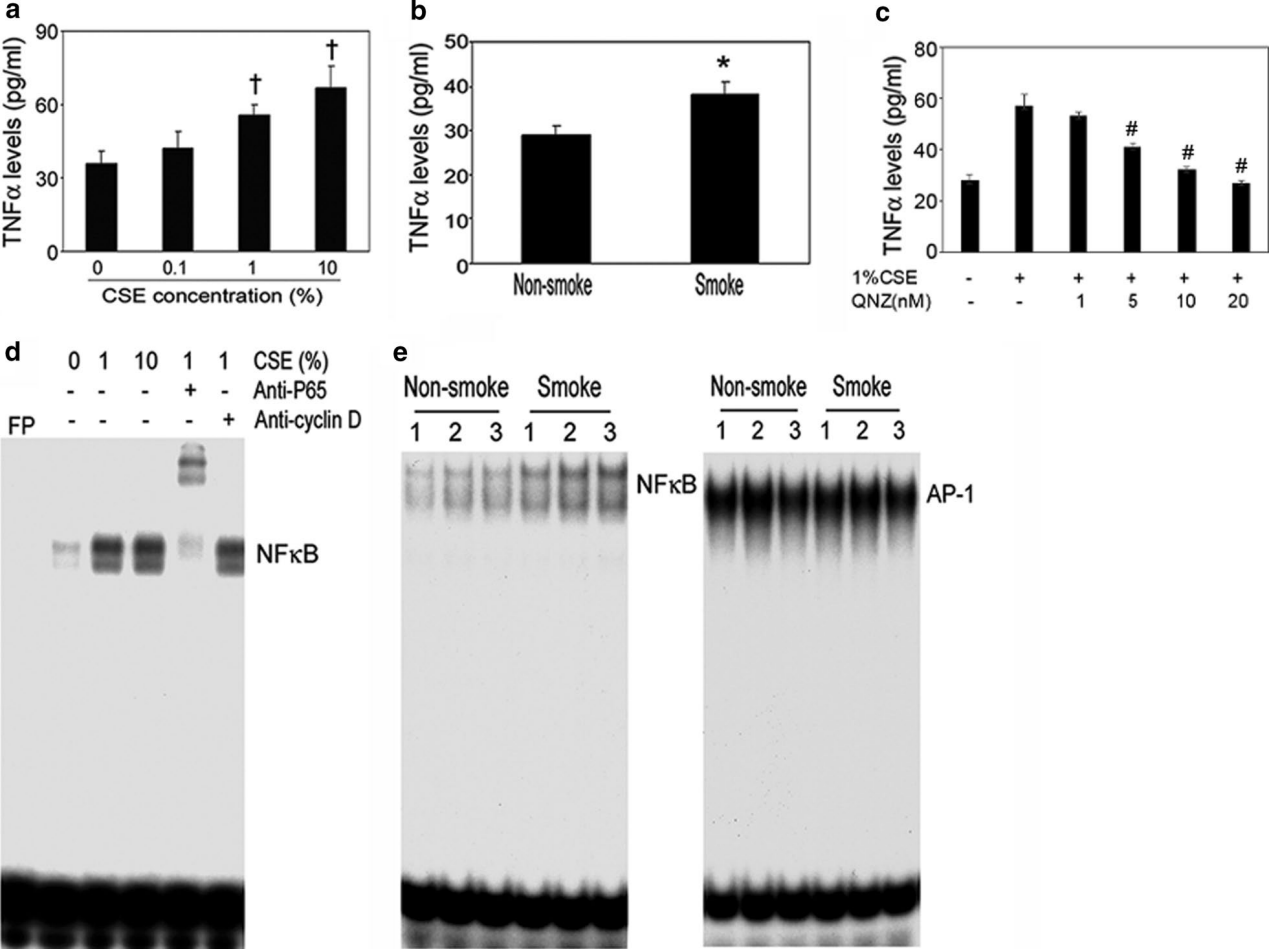
CSE impairs bone remodeling mediated by the NFκB pathway. The supernatants collected from differentiated BMSCs treated with CSE at concentrations as indicated. Bone marrow plasma collected from non-smoke and smoke-exposed mice. The TNFα levels of the supernatants (**a**) and bone marrow plasma (**b**) were measured by ELISA. Smoke exposure significantly increased TNFα expression. Consistently, CSE increased the levels of TNFα in BMSCs. **c** The supernatants collected from QNZ treated BMSC w/o 1% CSE. TNFα levels were significantly decreased. **b** Identification of DNA binding activity of CSE-regulated transcriptional factors using EMSA. The BMSC cells were treated with CSE, and nuclear proteins were extracted and incubated with [-^32^P]-labeled NFκB probe in the presence or absence of anti-*p65* antibody or anti-*cyclin D1* antibody. CSE induced the NFκB-p65 complex formation as evidenced by a gel shift using anti-*cyclin D1* antibody. **e** Nuclear proteins were extracted from BMSCs that were derived from non-smoke and smoke mice and incubated with labeled probes. Smoke exposure increased NFκB binding activity but not AP-1 binding activity. ^*^P < 0.05 compared to vehicle or non-smoke group; ^**^P < 0.001 compared to the vehicle; ^†,#^P < 0.01 compared to vehicle. All the data are from 3 independent experiments

To dispel the possibility that this induction of osteoclast differentiation and inhibition of osteoblast differentiation was solely the result of induction of inflammatory mediators in the bone marrow, we measured TNFα levels in the bone marrow adherent cells treated with CSE at different concentrations. CSE dose-dependently induced TNFα levels (Fig. 6a). The levels of TNFα in bone marrow plasma were significantly higher in the smoke-exposed animals than in the non-smoke-exposed animals (Fig. 6b). QNZ significantly inhibited TNFα production dose dependently (Fig. 6c). The involvement of NFκB was further demonstrated by NFκB inhibition in cells treated with CSE. These results suggest that TNFα could be one of the key mediators in the bone remodeling process altered by smoke exposure.

Aiming to identify the DNA binding activity of CSE-regulated transcriptional factors, we tested the subunits *p65* and *cyclin D1* by gel-shift assay. NF-κB controls cell growth and differentiation through transcriptional regulation of cyclin D1 [33]. We observed a shift due to the formation of the p65-NFκB complex, whereas no binding could be detected between the downstream target *cyclin D1* and NFκB, indicating that CSE affects NFκB signaling through complex formation (Fig. 6d). We also investigated the effects of smoking on the NFκB and AP-1 signaling pathways. Smoke exposure clearly induced NFκB expression in smoke-exposed animals compared to non-smoke-exposed animals, whereas no effect on AP-1 expression was detectable by gel-shift assay (Fig. 6e). These results distinctly indicate that smoke exposure induced RANKL activation, which is mediated by NFκB. Therefore, we propose that NFκB could be a "smoke-sensor" in bone cells.

## Discussion

The study intended to provide an in vivo mouse model of acute and chronic second-hand exposure to cigarette smoke. In particular, we performed a descriptive approach into the negative role of cigarette smoke induce inflammation on bone homeostasis. In the US, the total economic cost of smoking is more than $300 billion per year. According to the U.S. Department of Health and Human Services, this includes nearly $170 billion in direct medical care for adults, as well as more than $156 billion in lost productivity due to premature death and exposure to SHS. Smoking decreases life expectancy [34] and has been confirmed to be a causative factor in the acquisition of genetic lesions [35]. In the current study, we observed smoke exposure causing a significant imbalance between bone formation and bone resorption in both mouse models that were exposed to cigarette smoke for 10 days or 3 months. Ten-day smoke exposure efficiently induced osteoclast activity, inhibited osteoblast differentiation, and altered bone remodeling-related gene expression levels, although it did not transform the bone structure as drastically as their 3-month smoke exposure counterparts. Zhu, Sheng et al. reported recently (2021), they found similar results with our in vitro studies that bisphosphonates were able to reduce CSE-induced osteoporotic alternations using co-culture system [36].

The mechanisms by which smoking affects the skeleton may include both direct and indirect effects [37]. In an animal study, nicotine was shown to inhibit bone formation. In particular, a high concentration of nicotine profoundly inhibits oxidative metabolism and collagen synthesis in cultured chick bones [38]. In another study, a significant reduction of BMD was observed in nicotine-treated male mice and rats compared to animals without nicotine administration [39]. In consistence with our findings, there are a few groups reported in an i*n vivo* mouse model that animals exposed for 6 months to cigarette smoke has dramatically affect bone integrity and biomechanical properties [18, 40]. Based on a study of twin pairs discordant for cigarette smoking, women consuming one pack of cigarettes per day throughout life will have 5–8% less bone than non-smokers by the age of menopause [23]. Other researchers have reported 5–10% less bone and reduced protective effects of nutritional calcium in postmenopausal smokers than in nonsmokers [2, 41]. Of note, the duration of smoke exposure was negatively associated with BMD at the total hip, femoral neck, lumbar spine and total body in premenopausal women [42], confirming that postmenopausal women who smoke are at increased risk for osteoporosis. A cohort study suggests that compared with never smokers, current male cigarette smokers have an increased risk of hip fracture [4]. In the current study, we reported an approximate 24% decrease in bone volume in vertebrae and 23% decrease in trabecular BMD in tibiae of 3-month smoke-exposed animals. These profound changes found in mice were possibly due in part to the duration and dose of smoke exposure chosen in our protocols.

Tobacco smoke is a complex mixture of chemicals, each of which with the potential to act through different signal cascades, leading to the regulation of distinct transcriptional factors, such as NFκB, and subsequent alterations in gene expression [31]. In this study, we showed that activation of NFκB signaling pathways in bone cells by tobacco smoke exposure may be involved in the activation of resorption-induced genes such as RANKL, suggesting a potential mechanism for tobacco smoke-induced RANKL gene expression.

Given these changes in mRNA expression, we were interested in understanding whether the disparities arise from the alteration of mRNA transcription or translation. Studies towards a molecular understanding of inflammatory bone diseases have been highly successful from both an immunological and bone-centered perspective. The results of these studies led to the identification of several signaling pathways causally involved in inflammatory bone loss. Induction of RANKL signals by activated T cells and subsequent activation of the key transcriptional factors Fos/activator protein (AP-1), NFκB, and NF for activation of T cells c1 (NFATc1) are central to the signaling networks that to osteoclast-mediated bone loss [43].

Our data suggest that smoke exposure may also have a direct effect on bone. In support of our findings, it has been reported that in an in vivo mouse model, 2 weeks of smoke exposure stimulated the bone marrow cells and increased the size of the mitotic and postmitotic pools of polymorphonuclear leukocytes [44]. Mice exposed to cigarette smoke for three weeks exhibit loss of bone marrow B cells at the Pro-B-to-pre-B cell transition, with a largely reversed outcome after cessation of smoking 6 weeks [45]. Smoke exposure in immature rats significantly compromised bone health, leading to weaker bone [46]. There are other possible mechanisms for skeletal damage induced by smoking. It has been suggested that cigarette smoke may damage the blood supply and thus contribute to diminished bone strength [47]. In addition, cigarette smoke has been widely suggested to impact a broad range of immune functions [48, 49]. Finally, in support of our findings, Aspera-Werz, Romina et al. recently reported that extracts from conventional cigarettes significantly reduced the ALP activity and matrix mineralization at low concentrations [50].

In a prospective population-based cohort study of older men, people who stopped smoking have a significantly lower risk of hip fracture, at about 50%, during the first 10 years after stopping smoking [4, 51]. A decreased impact of smoking on the risk of hip fracture was also observed among women after cessation of smoking for more than 10 years [5]. Smoking cessation decreases *N*-terminal collagen cross-links (NTx) in postmenopausal women [52]. Additionally, smoking cessation is indicated as a preventive method and an effective measure to improve high-density lipoprotein functions in smokers suffering from coronary artery disease [53]. These studies suggest that the adverse effects of smoking are partially reversible. In future studies, it would be a high priority to further investigate whether the imbalance of bone remodeling caused by smoke exposure is reversible using a smoke cessation model.

We are cautious as to note that, in the current study, animal models are not completely replicate models of human cigarette smoking. In reality, smokers puff and then inhale in amounts that vary from moment to moment, and the amount drawn into the lungs differs from time to time, thus deviating from “standardized” machines used in animal experiments [54]. Other limitations for this report may include: a, the degree to which the particular concentrations and durations of smoke exposure used are biologically relevant; b, the use of CSE. CSE is advantageous in that it contains all the compounds inhaled by smokers, but due to the complexity of CSE, it is difficult to pinpoint which component mediates a specific effect. Nevertheless, we have begun to uncover the mechanisms of smoke exposure’s effects on bone remodeling.

## Supplementary Information

**Additional file 1: Fig. S1:**Ten-day smoke exposure did not significantly alter the bone structure in vivo. A. The parameters of bone phenotypes were measured by mictoCT in L5. B. Bone mineral density (BMD) was determined by pQCT in the tibiae.

## Data Availability

The data that support the finding of this study are available on request from the corresponding authors.

## Abbreviations

microCT: Micro computed tomography
pQCT: Peripheral quantitative computed tomography
NFκB: Nuclear factor kappaB
CS: Cigarette smoking
SHS: Second hand smoke
CSE: Cigarette smoke extract
TSP: Total smoke particulate
LPS: Lipopolysaccharide
BMD: Bone mineral density
AA: Ascorbic acid
TRAP: Tartrate-resistant acid phosphatase
ALP: Alkaline phosphatase
CatK: Cathepsin K
CTR: Calcitonin receptor
OPG: Osteoprotegerin
RUNX2: Runt-related transcription factor 2
OCN: Osteocalcin
Col I: Type I collagen
OSX: Osterix
BSP: Bone sialoprotein
OPN: Osteopontin
