# Supplementary material for: Cigarette smoke-associated inflammation impairs bone remodeling through NFκB activation
Source: J Transl Med. 2021 Apr 21;19:163. doi: 10.1186/s12967-021-02836-z (PMC8061040; doi:10.1186/s12967-021-02836-z)
Supplement: Supplementary file 1 — Additional file 1: Fig. S1:Ten-day smoke exposure did not significantly alter the bone structure in vivo. A. The parameters of bone phenotypes were measured by mictoCT in L5. B. Bone mineral density (BMD) was determined by pQCT in the tibiae. [file 12967_2021_2836_MOESM1_ESM.pdf]

Figure S1

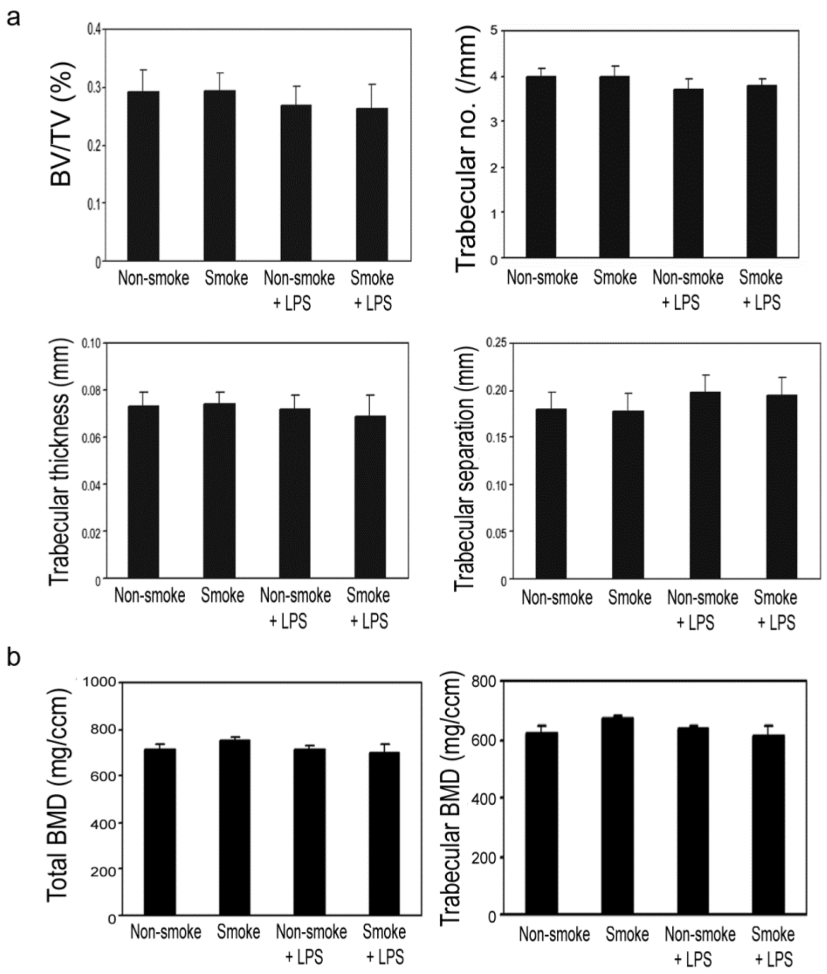

Fig. S1 Ten-day smoke exposure did not significantly alter the bone structure *in vivo*. a The parameters of bone phenotypes were measured by mictoCT in L5. b Bone mineral density (BMD) was determined by pQCT in the tibiae.
